# Ultra-dense, curved, grating optics determines peacock spider coloration

**DOI:** 10.1039/c9na00494g

**Published:** 2020-02-21

**Authors:** Bodo D. Wilts, Jürgen Otto, Doekele G. Stavenga

**Affiliations:** Adolphe Merkle Institute, University of Fribourg Chemin des Verdiers 4 CH-1700 Fribourg Switzerland bodo.wilts@unifr.ch; Grevillea Court 19 Grevillea Avenue St. Ives New South Wales 2075 Australia; Zernike Institute for Advanced Materials, University of Groningen NL-9747AG Groningen The Netherlands

## Abstract

Controlling light through photonic nanostructures is important for everyday optical components, from spectrometers to data storage and readout. In nature, nanostructured materials produce wavelength-dependent colors that are key for visual communication across animals. Here, we investigate two Australian peacock spiders, which court females in complex dances with either iridescent color patterns (*Maratus robinsoni*) or an approximately angle-independent blue coloration (*M. nigromaculatus*). Using light microscopy, FIB-SEM imaging, imaging scatterometry, and optical modeling, we show that both color displays originate from nanogratings on structured 3D surfaces. The difference in angle-dependency of the coloration results from a combination of the local scale shape and the nanograting period. The iridescence of *M. robinsoni* arises from ordered gratings on locally flat substrates, while the more stable blue colors of *M. nigromaculatus* originate from ultra-dense, curved gratings with multiscale disorder. Our results shed light on the design principle of the peacock spiders' scales and could inspire novel dispersive components, *e.g.* used in spectroscopic applications.

## Abstract 2

This study reports the optics of ultra-dense, nanoscopic gratings of peacock spiders that cause either angle-dependent or stable colours.

## Graphical abstract

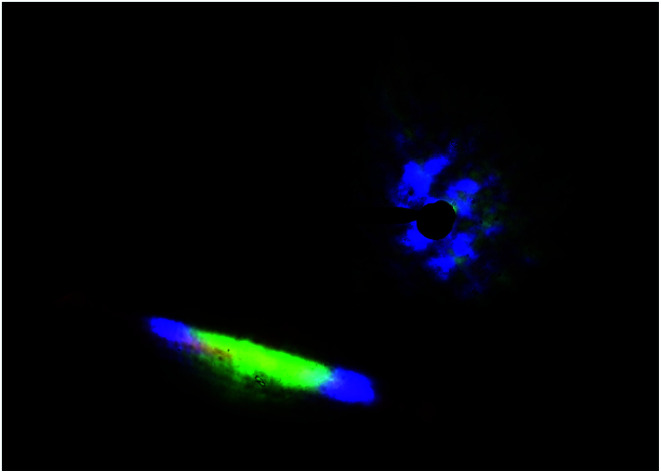

## Introduction

Nature has brought forward numerous physical solutions to interact with light, resulting in the splendid colors observed throughout the animal and plant kingdoms.^1–4^ A particularly colorful group of animals are the peacock spiders belonging to the genus *Maratus*, endemic to Australia.^5–7^ The males of these small, sexually dimorphic jumping spiders (body length 2–6 mm) are among the most brightly colored of the salticids.

Male peacock spiders are adorned with conspicuously colorful abdomens,^8,9^ whilst the females with a predominant brown/beige appearance are cryptically colored. During courtship rituals, a male peacock spider will raise his abdomen, and wave it side-to-side at a female in synchrony with his third pair of legs. Males of many *Maratus* species also have lateral flaps that can be extended from their abdomen like a fan. This fanning motion, together with the remarkable ornamentation of *Maratus* males, is reminiscent of a peacock's display, which has given the genus its common name.

The distinct color patterns observed across the various jumping spider species are produced by assemblies of tiny scales or hair-like protrusions, which reflect light in the visible and/or ultraviolet range.^10–13^ The optics of peacock spider scales is complex and intriguing and has just been started to be explored. So far, studies have found that the blue and green iridescent scales of *Maratus* males are mainly multilayer reflectors that produce interference-based colors,^10,11,13^ while the red and yellow patches of *Maratus* males arise from pigment-filled, brush-like scales.^13^ The elongated scales with diffraction gratings discovered in *Maratus robinsoni* and *M. chrysomelas* have inspired super-iridescent optics, but the biological samples itself have not been studied in detail.^12^

**Fig. 1:**
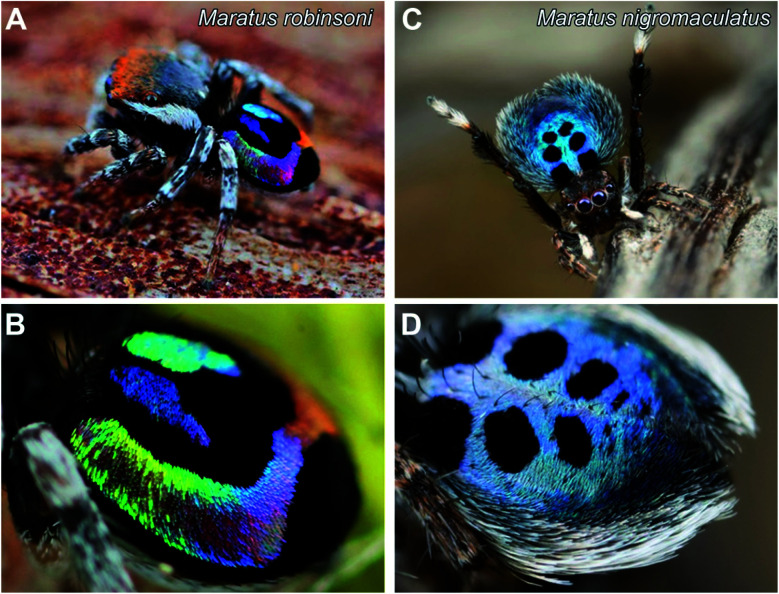
Peacock spiders with strongly colored abdomens. (A) Habitat photograph of an adult male *Maratus robinsoni*. (B) Enlarged view of the richly colored opisthosoma of *M. robinsoni*, contrasted by black areas. Note the swift color change of the colored areas. (C) Habitat photograph of an adult male *Maratus nigromaculatus*. (D) The opisthosoma has a blue angle-independent colour, with a pattern of black spots, surrounded by a white rim.

Here, we study the optics of the scales of two brilliant-colored and richly patterned peacock spiders, *Maratus robinsoni* and *M. nigromaculatus* (Fig. 1) using light microscopy, focused ion-beam scanning electron microscopy (FIB-SEM), imaging scatterometry, and optical modeling. We show that while the scales' appearances drastically differ in angle-dependency and color contrast, the optical mechanisms underlying their coloration, which rely on common grating interference, are surprisingly similar, with only minor structural adaptions to the local geometry.

## Results

### Appearance and optical properties

The male *M. robinsoni* (Fig. 1A and B) is a small (body length 2.5–3.0 mm), but very colorful spider. It has a nearly circular dorsal opisthosomal plate (flap/fan) with symmetric, large fields of vividly iridescent scales that reflect light directionally at wavelengths that span the visible spectrum. The colorful appearance is enhanced by a surrounding frame of jet-black scales. *M. nigromaculatus* (Fig. 1C and D) is slightly larger in size (body length 3–4 mm) and easy to recognize by the deep-blue flap, which carries six distinct, symmetrically arranged black spots, surrounded by a white-colored border.

**Fig. 2:**
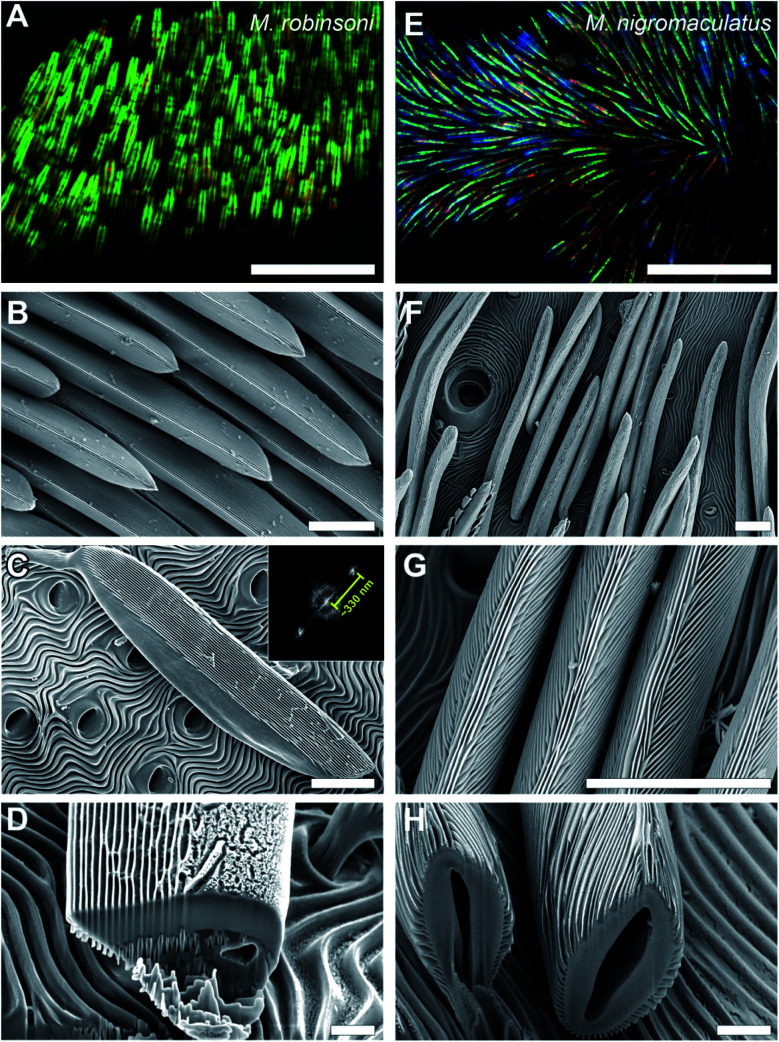
Optical and electron micrographs of the abdominal scales of *M. robinsoni* and *M. nigromaculatus*. (A and E) Optical micrographs showing the more or less parallel alignment of the scales. (B–H) SEM micrographs showing that the scales' surface has a grating structure. (B–D) The grating on the wedge-shaped scales of *M. robinsoni* is arranged parallel to the long axis of the scale with a mean period of ∼330 nm (inset of C). (F–H) The grating of *M. nigromaculatus* scales curves along the cylindrical scale. Scale bars: (A and E) 50 μm, (B, C, F and G) 10 μm, (D and H) 2 μm.

The prominent colors of both spider species originate in hair-like scales imbricating the flap in more or less straight lines (Fig. 1 and 2). Observed with high magnification under a light microscope, the hair-like scales of *M. robinsoni*, green with an orange stripe along its center, lay parallel on the flap, above a deep black cuticle (Fig. 2A). SEM images confirm the parallel arrangement on the flap (Fig. 2B). The cylindrical scales have a complex 3D shape with a sharp edge, created by two angled planes, which have a grating-like structure (Fig. 2C and D). These sharp edges are facing away from the body when the scales are mounted on the flap. A side-view of a tilted hair shows a regular array of protrusions that run parallel to the long edge of the hair (Fig. 2C). Indeed, an FFT transform of the image shows a highly symmetric pattern (inset of Fig. 2C) with a periodicity of about 330 nm (Table 1). To reveal the 3D shape of the scales, we performed FIB-SEM and gently milled a scale to expose the cross-section. Clearly, the upperside of the scale is wedge-shaped with a symmetric grating on both sides above a spherical underside that is void of the grating structure (Fig. 2D). The wedge is ∼7 μm high and has a base of ∼6 μm, resulting in an angle of ∼50° between the sides that carry the gratings. Only a single grating periodicity was observed in our samples, contrary to previous observations.^12^

**Table 1:** Grating parameters of *M. robinsoni* (*N* = 19) and *M. nigromaculatus* (*N* = 17) scales

Species	Width of grating	Distance of grating	Effective grating
*M. robinsoni*	140 ± 20 nm	330 ± 15 nm	3000 lines per mm
*M. nigromaculatus*	80 ± 20 nm	210 ± 30 nm	4600 lines per mm

Similar to *M. robinsoni*, the blue scales of *M. nigromaculatus* lay on the flap, above a deep black cuticle (Fig. 2E), though without the parallel arrangement observed in *M. robinsoni*. SEM images reveal that the scales of *M. nigromaculatus* are sparsely and disorderly arranged on the flap and have a complicated surface pattern (Fig. 2F). A grating structure is observed, but it here curves along the scale's long axis (Fig. 2G). Compared to *M. robinsoni*, this grating is much denser and features a mean period of ∼210 nm (Table 1). The scales have a hollow center and the grating is present along their entire circumference (Fig. 2H).

**Fig. 3:**
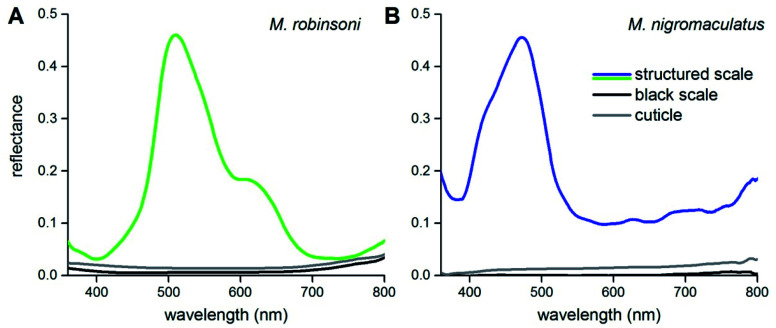
Reflectance spectra of scales and cuticles measured with a microspectrophotometer. (A) *M. robinsoni*. (B) *M. nigromaculatus*.

To show that the scale gratings create the different intense colors, we measured reflectance spectra of the peacock spider scales with a microspectrophotometer (Fig. 3). Reflectance spectra of single iridescent scales of *M. robinsoni* have an asymmetric shape with a peak at ∼510 nm and a shoulder at ∼600 nm (Fig. 3A, green line). The reflectance of the cuticle is very minor (Fig. 3A, gray line), and the reflectance of black scales is even much less: <1% over the whole visible wavelength range (Fig. 3A, black line). The blue scales of *M. nigromaculatus* have a distinct reflectance band peaking at ∼470 nm (Fig. 3B, blue line). Here, the cuticle and black scales reflect even less than those of *M. robinsoni*; the black scale reflectance was close to the detection limit of our system (<0.2%) over the whole visible wavelength range (Fig. 3B, black line).

### Light scattering of single scales

**Fig. 4:**
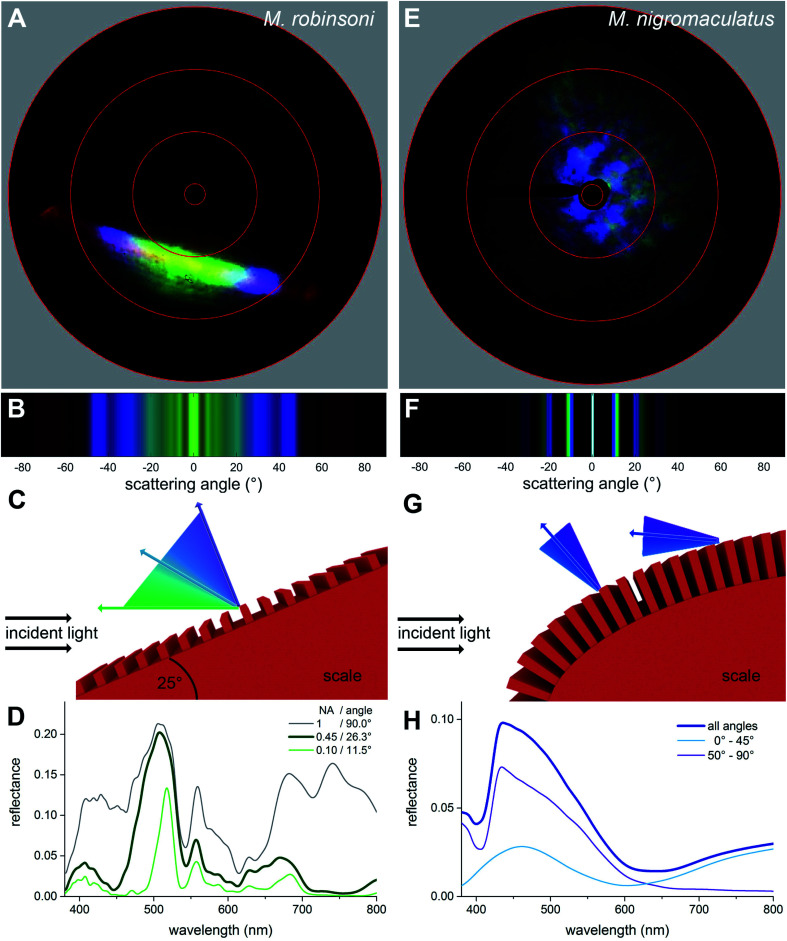
Imaging scatterometry and optical modelling of the grating structures. (A and E) Scatterograms of *M. robinsoni* and *M. nigromaculatus* scales, obtained with local illumination. (B and F) Optical modelling of the diffraction pattern of the grating structures. (C and G) Sketches of the reflection mechanism for both scales. (D and H) Modelled reflectance spectra as a function of the detection aperture (D) and illumination angle (H).

To investigate why the flaps of *M. robinsoni* are iridescent, with strongly varying colors dependent on the direction of illumination and viewing angle, while the flaps of *M. nigromaculatus* display a virtually identical blue color when illuminated or observed from any direction, we performed imaging scatterometry on single scales (Fig. 4A and E). For this, small pieces of the abdominal flaps were glued to the tip of a glass micropipette and mounted in the imaging scatterometer.^14,15^ Light was then focused on a single hair with a spot size of ∼13 μm.

Scatterograms from single *M. robinsoni* scales show that incident light is diffracted into a highly restricted spatial angle (about a plane, appearing in the scatterogram as a line), with a strong color change in the direction perpendicular to the scale's longitudinal axis, *i.e.*, to the grating (Fig. 4A). Yellow-greenish light is reflected in the normal direction, *i.e.* in the center of the diffraction line, while the color is progressively blue-shifted at higher scattering angles; purple light is reflected into a scattering angle of ∼45°, while reddish light appears at scattering angles above 60°. Very differently, as shown by the scatterogram of a single *M. nigromaculatus* scale, blue light is reflected diffusely around the specular angle direction of the incident beam, with distinct lines close to this central, direct reflection (Fig. 4E).

### Spectral modeling of ultra-dense diffraction gratings

To understand the optics of the flat grating of *M. robinsoni vs.* the curved grating of *M. nigromaculatus* scales, and especially the influence of the curvature of the latter grating on the light scattering of the scales, we modeled the gratings using both common grating optics and finite-difference time-domain (FDTD) simulations. In our first calculations, we assumed a flat grating and thus used the reflection grating equation given by

1 *mλ*_g_ = *d*(sin *θ*_in_ + sin *θ*_out_)

with the grating order *m* for wavelength *λ*_g_, grating period *d*, and incident and diffracted angles *θ*_in_ and *θ*_out_, respectively.

We modeled the *M. robinsoni* grating by taking a repetitive grating of ∼3.000 lines per mm, with light normally incident on the scale with top angle 50°, *i.e.* the incident angle on the grating was (90 − (50/2))° = 65° (Fig. 4C). For this scenario, the forward scattering of the 0^th^ order is reflected towards the black cuticle and will be absorbed by it.^7^ Consequently, only the −1^st^ order is reflected back into the direction of the observer and the angle-dependent behavior is described by eqn (1) (Fig. 4C). A simulated far-field scattering pattern of the *M. robinsoni* morphology for normal-incident light on a grating-carrying prism (Fig. 4B) is very similar to the measured scatterogram of Fig. 4A. Yellow-green light is reflected towards the observer, while blue-violet light gets diffracted into higher angles; at large angles, above 65°, a faint reddish reflection appears (Fig. 4B and C). This reversed color sequence with respect to a conventional diffraction grating (from green to blue rather than blue to green for higher angles, Fig. 4B) is a direct consequence of the shape of the hair. The prismoidal shape keeps the diffraction grating under a more vertical alignment and results in this reverse order while the grating at normal incidence still works as a common diffraction grating (see sketch in Fig. 4C), as previously described for biological and bio-inspired systems with a reverse color sequence.^12,16^Fig. 4D shows simulated reflectance spectra for the full 3D structure at normal incidence for a varying numerical aperture (NA) of the detector. The spectrum for NA = 0.45 qualitatively agrees well with the experimentally measured spectrum of Fig. 3A concerning peak position and the appearance of a shoulder. These simulations were performed on an idealized structure, and so did not include the subtle variations in shape and grating period of the extant gratings that will cause a spectral broadening. A (hypothetical) optical system that could sample the whole hemisphere would measure significantly more red light (Fig. 4D, gray line). The spectrum for NA = 0.1 (Fig. 4D, green line) shows the aperture-dependent filtering performance of the grating.

*M. nigromaculatus* with its ultra-dense grating (∼4600 lines per mm) that curves along the length of the scale has a very different scattering pattern (Fig. 4E). A grating of this density reflects preferably blue light (Fig. 4F–H). Due to the curvature of the scale, the incidence angle varies locally on the grating, so that blue light is reflected in multiple spatial directions, most intense close to the center (Fig. 4E and F). It follows from eqn (1) (see also ref. 17) that the grating has a cut-off wavelength at around 480 nm, meaning that for wavelengths above 480 nm no wavelength is diffracted outside the 0^th^ (specular) order. This makes this grating an effective low-pass filter that only supports UV-blue light. The local curvature of the grating on the scale further diminishes possible angle-dependent effects. Additionally, the ellipsoidal shape of the hair, due to its variation in local curvature, effectively superimposes the diffraction patterns created by different possible incidence angles, resulting in a diffuse, angle-independent blue scattering pattern.

We subsequently performed FDTD-simulations of a flat diffraction grating under different incident angles to mimic the curvature of the curved hair. The calculated reflectance spectra of Fig. 4H support the grating calculations. An average of the calculated reflectance spectra over all incidence angles limited by the aperture of the microscope objective used in the MSP measurements (Fig. 4H, dark blue curve) corresponds reasonably well with the experimental spectrum of Fig. 3B. Indeed, blue light is reflected towards the observer for all angles of light incidence, though at large incidence angles the blue-peaking reflectance is more pronounced with an added UV component (Fig. 4H, purple line).

## Discussion

Spiders employ a rich variety of structural coloration mechanisms, ranging from common multilayered structures,^10,11,13^ to coaxial Bragg mirrors,^18,19^ to the nanogratings observed in the peacock spiders as well as in other spiders.^20^ The investigated peacock spiders feature a common coloration motif in the form of an ultra-dense diffraction grating (Fig. 2). We demonstrate here that changes of the local grating period and scale curvature highlight how topological variations combined with a denser grating array can result in colors with strongly different visual appearances: from the strongly iridescent colors of *M. robinsoni* to the colors with virtually no angle-dependency of *M. nigromaculatus*.

In addition to just these topological variations, the scales of *M. nigromaculatus* also feature multi-scale disorder. Disorder can be observed in the macroscopic arrangement of the scales on the flap (Fig. 1C, D and 2E), and local microscopic disorder occurs due to the curvature of the grating along the scale (Fig. 2G and H) and a slightly different grating period on top (Fig. 2F and G). We expect that this disorder will spread out the optical signal created by the grating and further enhance the angle-independence, similar to disordered gratings observed in flower petals.^21^

*Maratus* spiders are extremely visual animals, where the colored flaps play an important role in elaborate courtship rituals,^5,6,12,13^ next to other factors, as odors.^22^ Male *M. robinsoni* create a strongly dynamic, time- and spectral-dependent, iridescent signal, quite similar to the deeply colored feathers of the bird-of-paradise Lawes' parotia.^23,24^ In both cases, a complex 3D shaped reflector causes an iridescent display. Quite in contrast is the blue color of male *M. nigromaculatus* spiders (Fig. 1). In this species, the curved, ultra-dense grating results in a nearly diffuse reflection of a constant blue color (Fig. 4). In other animals, non-iridescent blue colors are, for example, achieved by more complex structures featuring significant amounts of disorder.^18,21,25–27^

How the different dynamic signals radiated by the male spiders are perceived by the females remains to be investigated. We note here that the eyes of jumping spiders have a very high spatial and temporal acuity,^28,29^ possibly paired with tetrachromatic vision,^13,30,31^ making it highly likely that female spiders are able to perceive the males' dynamic coloration during their elaborate courtship behavior.

Nature's unique solutions to optical problems have since long stimulated bio-inspired applications.^32–34^ Especially, light control by gratings is used in everyday life, ranging from data readout/storage to spectrometers.^12,17^ However, ultra-dense gratings as the ones found on *M. nigromaculatus* scales are difficult to manufacture and are technologically so far only used in deep-UV applications due to their “poor” performance and applicability, particularly as the cut-off wavelength of these gratings lays in the visible wavelength range.^35^ It is noteworthy that the male *M. nigromaculatus* employs this spectral cut-off behavior of such ultra-dense nanogratings to create a stable blue color (Fig. 4), quite different from other known ways to create spider blues.^13,18^

The design of gratings for nanoscopic, small-scale applications is still a challenge. Our identified design parameters for nanoscopic gratings and the influence of the local topology on the selective dispersion of incident light should provide a source of inspiration for designing further dispersive elements, with impact in the field of optical sciences.

## Experimental section

### Samples

Male *M. robinsoni* (Otto and Hill, 2012) and *M. nigromaculatus* (Keyserling, 1883) were locally captured in New South Wales (*M. robinsoni*) and Queensland (*M. nigromaculatus*), Australia. All specimens were preserved in 70% ethanol. Details of both species' distribution can be found in ref. 6, 8 and 9. Survey images of the scale organization at the opisthosomal flaps were made with an Olympus SZX16 stereomicroscope (Olympus, Tokyo, Japan) or a Zeiss Universal Microscope (Zeiss, Oberkochen, Germany) equipped with an Olympus LUCPlanFL N 20×/0.45 objective. All measurements were performed on at least two different specimens.

### Spectroscopy

Reflectance spectra of single scales *in situ* and of bare cuticle (Fig. 3) were measured with a microspectrophotometer (MSP), being a Leitz Ortholux microscope (Leitz, Wetzlar, Germany) connected to an AvaSpec 2048-2 CCD detector array spectrometer (Avantes, Apeldoorn, The Netherlands), with light supplied by a xenon arc light source. The microscope objective was an Olympus LUCPlanFL N 20×/0.45. A white diffuse reference tile (Avantes WS-2) was used as a reference.

### Imaging scatterometry

The far-field spatial reflection characteristics of the scales were studied with an imaging scatterometer. A small piece of cuticle with scales attached was glued to the tip of a glass micropipette. Scatterograms were obtained by focusing a white-light beam with a narrow aperture (less than 5°) onto a small circular area (diameter ∼ 13 μm) of a scale, and the spatial distribution of the far-field scattered light was then monitored. A flake of magnesium oxide served as a white diffuse reference object; for further details see ref. 14 and 15.

### Anatomy

Opisthosomal flap pieces were cut, glued onto fitting stubs, and sputtered with a 5 nm thick layer of gold. The ultrastructure was observed with a MIRA 3 LMH field-emission electron microscope (Tescan, Brno, Czech Republic). For ultrastructural investigations, we performed focused-ion beam milling of the scales using a FEI Scios 2 (FEI, Eindhoven, The Netherlands) dual beam field-emission electron microscope equipped with a gallium-ion ion beam (operated at 30 kV, 0.3 nA).

### Optical modelling

The light scattering of the ultrastructure was simulated using the finite-difference time-domain (FDTD) method for different grating parameters using Lumerical FDTD. The ultrastructure was approximated with a periodic, dielectric grating (Table 1) of chitin and the incidence angle of light was altered. The wavelength range was limited to 360–800 nm. The simulated far-field scattering pattern was transformed into CIE1976 color space using a custom-written routine in Matlab.

## Conflicts of interest

There are no conflicts to declare.

## Supplementary Material
